# Dual-actuator-type active noise control in vibro-acoustic systems with openings

**DOI:** 10.1038/s41598-025-17810-8

**Published:** 2025-09-02

**Authors:** Khaled Said Ahmed Maamoun, Chung Kwan Lai, Stanislaw Wrona, Marek Pawelczyk, Hamid Reza Karimi

**Affiliations:** 1 Department of Measurements and Control Systems, Silesian University of Technology, Gliwice, 44-100 Poland; 2 Institute of Sound and Vibration Research, University of Southampton, Southampton, SO17 1BJ UK; 3 Department of Mechanical Engineering, Politecnico di Milano, Milan, 20156 Italy

**Keywords:** Active noise control, Active structural acoustic control, Virtual sound barrier, Vibrating plates with openings, Sound power reduction, Mechanical engineering, Electrical and electronic engineering

## Abstract

Openings in plate structures are essential in various engineering applications, particularly in vibro-acoustic systems where airflow is required. This paper investigates noise control in vibro-acoustic systems with noise barriers incorporating structural openings, focusing on active noise control and Active Structural Acoustic Control (ASAC). It also introduces a novel approach, Dual-Actuator-Type Active Noise Control (DATANC), which combines loudspeakers and inertial actuators into the same barrier to address the challenges of noise reduction. A sound power estimation method is proposed to account for sound transmission through the opening and is integrated into an analytical model for optimizing actuator placement; predictions show strong agreement with observed behavior. Among the ASAC configurations, experimental analysis shows that actuators placed near the edge of the opening achieve the greatest noise reduction in the 100–200 Hz range, where acoustic leakage is dominant. DATANC consistently outperformed all single-actuator configurations, delivering superior attenuation of dominant vibro-acoustic resonances while maintaining reasonable computational complexity. The analysis is extended to a plate with a transparent lid over the opening to evaluate the contribution of acoustic leakage to the system performance. The findings of this study demonstrate that optimized actuator placement, combined with DATANC, provides a practical solution for noise control in systems where structural openings are required.

## Introduction

The rapid development of industrial equipment, household devices, and the automotive and transportation industries has significantly contributed to the rise in global noise pollution levels. Due to its significant impact on both physiological and psychological health, extensive research efforts have focused on mitigating noise levels. Openings in the plate structure of vibro-acoustic systems serve essential functions, including heat dissipation, routing electrical cables, providing personnel access, and weight reduction^1,2^. However, the shape and location of the openings, as well as their relative position to the noise source in such systems, have a significant impact on the system’s sound power response^3^. Significant effort has been made by researchers to control the sound power characteristics of vibro-acoustic systems. Passive noise control (PNC) provides solutions that do not require an external energy supply, typically achieving optimal noise reduction through modifications to the physical properties of the barriers separating the noise source from the surroundings^4,5^. PNC is effective for noise attenuation in a considerably high-frequency range compared to active noise reduction strategies, which are generally more effective below this range^6^. Passive noise barriers are typically thick, heavy, and often provide significant heat insulation, which can create additional challenges in certain applications. As a result, actively controlled barriers present a more suitable alternative. These barriers integrate control sources, which may be acoustic, such as loudspeakers, or structural, such as inertial actuators^7^.

Based on the concept of canceling noise using sound waves of equal amplitude and opposite phase, Active Noise Control (ANC) was first proposed and patented by Lueg in 1936^8^. Since then, ANC systems have become essential tools for noise reduction in various engineering applications, including automotive cabins, aircraft interiors, and domestic appliances^9–12^. The filtered-x least-mean-square (FxLMS) algorithm is the most popular ANC algorithm due to its stability and simple structure as the reference signal to the controller is filtered through the model of the secondary path between the actuator and the error sensors ^13,14^. In practice, the output amplitude of the control filter must be constrained to limit the maximum output level and prevent actuator overload^15,16^. Thus, the leaky FXLMS algorithm is one of the methods used to limit output power by introducing a leakage factor that constrains the control effort and stabilizes the adaptation process^17,18^. ANC is also employed to establish a Virtual Sound Barrier (VSB) in enclosed systems with openings. Secondary actuators can be placed along the boundary of the opening, a technique referred to as boundary control^19^. This approach allows for improved airflow since the speakers do not obstruct the opening. Nonetheless, the boundary length can impose physical constraints on controllability and requires sufficient thickness to mount the actuators securely. In the contrary, planar control creates a grid of the secondary actuators on the opening plane which limits the accessibility to the noise source^20,21^. ANC has been shown to achieve up to a 10 dB reduction in broadband urban noise through a fully opened window^22^. However, placing a planar loudspeaker array directly across the opening may obstruct accessibility. Additionally, ANC can be combined with micro-perforated panel absorbers (MPPA) to enhance broadband noise reduction in enclosed spaces where high sound energy dissipation is required^23^.

On the other hand, Active Structural Acoustic Control (ASAC) is typically implemented using passive noise barriers, vibration actuators, sensors, and a control system^24,25^. It’s a well-established technique for reducing noise and structural vibrations. With careful implementation, ASAC can achieve not only local noise attenuation but also global noise reduction^26,27^. ASAC was implemented in a closed passenger car equipped with an active windshield, achieving up to 15 dB reduction in interior sound pressure levels. The study focused on a fully sealed cabin, without addressing the effects of openings such as partially open windows on acoustic performance^28^.The performance of a tonal ASAC strategy that utilizes an experimentally identified radiation matrix to control sound power radiation was investigated, demonstrating superior performance compared to the active vibration control method^29^. The study was limited to tonal excitation, with broadband disturbances not addressed. A feedforward ASAC algorithm was also tested on the lining panels of an aircraft cabin using a low-cost microcontroller, achieving a tonal noise reduction of 23 dB^30^. A radiation-mode-based ASAC system for an enclosure with vent openings has been theoretically evaluated^31^.However, the model was validated using COMSOL-based finite element (FE) and boundary element (BE) simulations, and the analysis was limited to tonal disturbances. Existing studies reveal a gap in ASAC research on systems with openings compared to fully enclosed systems, highlighting the need for further investigation. The placement of inertial actuators on vibrating plates is a critical factor influencing system performance. When properly positioned, actuators can significantly enhance both the vibration and acoustic responses. Various studies have explored actuator placement optimization using cost functions derived from vibration characteristics^32,33^ and modal radiation analysis^34^. Although the active force of inertial actuators can be evaluated using linear quadratic optimal control theory applied to analytical models^35,36^, these approaches do not account for systems with openings, which introduce additional complexity due to the acoustic leakage.

This paper introduces the Dual-Actuator-Type Active Noise Control (DATANC) system, which combines loudspeaker and inertial actuators to address noise reduction challenges in vibro-acoustic systems with openings. To investigate its effectiveness, the DATANC system is experimentally evaluated alongside conventional ANC and ASAC configurations with a single type of actuators. The present study experimentally evaluates ASAC in these systems under random noise excitation, extending beyond prior work focused on tonal disturbance excitation^31^. To enable a fair comparison between different control configurations, the locations of the inertial actuators are optimally determined using an analytical model that accounts for sound transmission through the enclosure opening. This approach builds on previously established control strategies^35,36^. Additionally, the study evaluates the impact of this acoustic leakage on the overall sound power response by analyzing a vibrating plate fitted with a transparent lid over the opening. The system, with a transparent lid, itself serves as an effective solution to enhance noise barrier functionality. Nevertheless, the transparency of the material allows for continued device monitoring. The main contributions of this paper are as follows: i) the optimal placement of inertial actuators using an analytical model that accounts for sound transmission through the enclosure opening; ii) the development of the DATANC system, which integrates loudspeakers and inertial actuators to achieve enhanced noise control performance in these systems, outperforming conventional ANC and ASAC configurations under broadband random noise excitation; iii) a spatial analysis of the relative placement of actuators and its influence on system performance, including both frequency response behavior and control effectiveness; iv) an investigation of the acoustic transmission effect by introducing an enclosure opening patched with a transparent lid.

## Methods

**Fig. 1:**
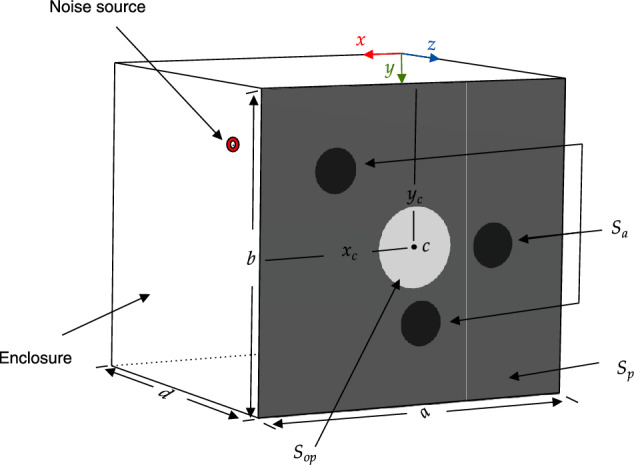
Analytical model configuration showing the enclosure domain, the vibrating plate $S_p$, a circular opening interface $S_{op}$ with a radius of $0.05~m$, and the actuators domain, $S_a$, is a combination of circles with a radius of $0.02~m$ for each actuator. The position $(x_{c}, y_{c})$ is the center of $S_{op}$ and the primary noise source is positioned at $(x_{s} = 0.3~m, y_{s} =0.1~m, z_{s}=-0.2~ m)$.

A previously introduced vibro-acoustic system^1^ is modified to incorporate inertial actuators, treating the vibrating plate as an actively controlled acoustic barrier. This modification is primarily aimed at enabling the optimal placement of inertial actuators by accounting for both radiated sound and acoustic transmission through structural openings. The system consists of a rectangular enclosure with a freely vibrating, fully clamped rectangular plate that includes a circular opening interface, as illustrated in Fig. 1. The remaining five walls of the enclosure are modeled as acoustically rigid (sound hard).

### Analytical model

The Kirchhoff–Love theory of thin plates^37^ is employed to model the vibrating plate in free space, under the assumptions of isotropy and homogeneity. The analysis considers only transverse motion, while the effects of rotary inertia are neglected. The kinetic energy of the plate, $T_{p}$, is expressed as:

1 $$\begin{aligned} T_{p} = \frac{1}{2} h \iint _{S_{mix}} \rho (x,y)\, \dot{w}^2 \, \textrm{d}x\, \textrm{d}y, \end{aligned}$$

where $h$ is the thickness of the plate, and $\rho (x,y)$ represents the varying density of the mixed interface, $S_{mix}$. The density function is defined as:

2 $$\begin{aligned} \rho (x, y) = {\left\{ \begin{array}{ll} \rho _p, & \text {if } (x, y) \in S_{p}, \\ \rho _{act}, & \text {if } (x, y) \in S_{a}, \\ 0, & \text {if } (x, y) \in S_{op}, \end{array}\right. } \end{aligned}$$

where $\rho _p$ is the density of the plate material, and $\rho _{act}$ is a theoretical density value representing the mass effect of the actuators on the system kinetic energy. The actuators, being small relative to the dimensions of the plate, have a negligible effect on the strain of the plate surface. By assuming ideal bonding and neglecting the stiffness of the actuators^34,38,39^, the total energy contribution from the actuators can be represented by $\rho _{act}$ in the plate kinetic energy function, as in Eq. (1, 2). The deflection of the plate in the $z$-direction at time $t$ is denoted as $w(x, y, t)$. The initial conditions are given by

3 $$\begin{aligned} w(x, y, t_0) = 0 , \quad \frac{\partial w(x, y, t)}{\partial t} \bigg |_{t = t_0} = 0, \quad t \ge t_0 > 0. \end{aligned}$$

### Sound power estimation

The estimation of sound power is based on radiation from the vibrating plate and the sound transmission through the opening. The total sound power is given by

4 $$\begin{aligned} W_{\text {total}}(\omega ) = W_{\text {rad}}(\omega ) + W_{\text {loss}}(\omega ), \end{aligned}$$

where $W_{\text {rad}}(\omega )$ is the radiated sound power from the plate and can be expressed in vector form as

5 $$\begin{aligned} W_{\text {rad}}(\omega ) = q_r^H \textbf{R}(\omega ) q_r, \end{aligned}$$

where the superscript *H* denotes the complex conjugate transpose. The vector $q_r$ represents the plate velocity and is obtained from the last $n_p^2$ elements of the system response vector *q*. Here, $n_p$ is the number of modes considered in one direction of the 2D vibrating plate, and $\textbf{R}(\omega )$ is the radiation resistance matrix at frequency $\omega$, computed analytically using surface discretizations and mutual element interactions ^1,36,40^. $W_{\text {loss}}(\omega )$ is the leakage sound power and can be estimated as follows:

6 $$\begin{aligned} W_{\text {loss}}(\omega ) = \frac{q_p^H q_p}{\rho _a c} A_s. \end{aligned}$$

Where $q_p$ is the pressure at the opening interface and can be calculated from the first $n_c^3$ elements from the system response, $n_c$ is the number of modes considered in one direction of the 3D enclosure, $A_s$ is the element area, $\rho _a$ is the density of the air, and *c* is the speed of sound in the air. By substituting Eqs. (5, 6) in Eq. (4), the total sound power in vector form is expressed as

7 $$\begin{aligned} W_{\text {total}}(\omega )= q_r^H \mathbf {R(\omega )}q_r + \frac{q_p^H q_p}{\rho _a c} A_s. \end{aligned}$$

Based on the additive theory ^41^, the plate velocity $q_r$ can be expressed as

8 $$\begin{aligned} q_r = q_{r,\text {pri}} + \sum _{k=1}^K q_{r\_\text {unit},k} f_{c,k}, \end{aligned}$$

where $q_{r,\text {pri}}$ is the velocity due to the primary source, $q_{r\_\text {unit},k}$ is the velocity due to the *k*-th unit control source, and $f_{c,k}$ is the complex amplitude of the *k*-th control source. Similarly, the pressure $q_p$ at the opening interface can be written as

9 $$\begin{aligned} q_p = q_{p,\text {pri}} + \sum _{k=1}^K q_{p\_\text {unit},k} f_{c,k}, \end{aligned}$$

where $q_{p,\text {pri}}$ is the pressure due to the primary source, and $q_{p,\text {unit},k}$ is the pressure due to the *k*-th unit control source. The quantities $q_{r\_\text {unit},k}$ and $q_{p\_\text {unit},k}$ are unique for each actuator as they differ due to the different location of the actuators on the plate. Substituting the expansion of $q_r$ into the radiated sound power $W_{\text {rad}}(\omega )$, we obtain:

10 $$\begin{aligned} \begin{aligned} W_{\text {rad}}(\omega )&= q_{r,\text {pri}}^H \mathbf {R(\omega )} q_{r,\text {pri}} + \sum _{k=1}^K \sum _{j=1}^K f_{c,k}^H q_{r,\text {unit},k}^H \mathbf {R(\omega )} q_{r,\text {unit},j} f_{c,j} \\&\quad + \sum _{k=1}^K f_{c,k}^H q_{r,\text {unit},k}^H \mathbf {R(\omega )} q_{r,\text {pri}} + \sum _{k=1}^K \big (q_{r,\text {unit},k}^H \mathbf {R(\omega )} q_{r,\text {pri}}\big )^H f_{c,k}. \end{aligned} \end{aligned}$$

For the leakage sound power $W_{\text {loss}}(\omega )$, substituting the expansion of $q_p$, we get:

11 $$\begin{aligned} \begin{aligned} W_{\text {loss}}(\omega )&= \frac{1}{\rho _a c} A_s \Bigg ( q_{p,\text {pri}}^H q_{p,\text {pri}} + \sum _{k=1}^K \sum _{j=1}^K f_{c,k}^H q_{p,\text {unit},k}^H q_{p,\text {unit},j} f_{c,j} \\&\quad + \sum _{k=1}^K f _{c,k}^H q_{p,\text {unit},k}^H q_{p,\text {pri}} + \sum _{k=1}^K \big (q_{p,\text {unit},k}^H q_{p,\text {pri}}\big )^H f_{c,k} \Bigg ). \end{aligned} \end{aligned}$$

Hence, the total sound power can be expressed as

12 $$\begin{aligned} \begin{aligned} W_{\text {total}}(\omega )&= q_{r,\text {pri}}^H \mathbf {R(\omega )} q_{r,\text {pri}} + \frac{q_{p,\text {pri}}^H q_{p,\text {pri}}}{\rho _a c} A_s + \sum _{k=1}^K \sum _{j=1}^K f_{c,k}^H q_{r,\text {unit},k}^H \mathbf {R(\omega )} q_{r,\text {unit},j} f_{c,j} \\&\quad + \sum _{k=1}^K f_{c,k}^H q_{r,\text {unit},k}^H \mathbf {R(\omega )} q_{r,\text {pri}} + \sum _{k=1}^K \big (q_{r,\text {unit},k}^H \mathbf {R(\omega )} q_{r,\text {pri}}\big )^H f_{c,k} \\&\quad + \frac{1}{\rho _a c} A_s \Bigg ( \sum _{k=1}^K \sum _{j=1}^K f_{c,k}^H q_{p,\text {unit},k}^H q_{p,\text {unit},j} f_{c,j} + \sum _{k=1}^K f_{c,k}^H q_{p,\text {unit},k}^H q_{p,\text {pri}} + \sum _{k=1}^K \big (q_{p,\text {unit},k}^H q_{p,\text {pri}}\big )^H f_{c,k} \Bigg ). \end{aligned} \end{aligned}$$

Using the linear quadratic optimal control theory, we minimize $W_{\text {total}}(\omega )$ by setting it’s gradient to zero. The optimal gain $f_{c,k}^\text {opt}$, can be calculated by solving the linear system:

13 $$\begin{aligned} \textbf{f}_c^\text {opt} = -\textbf{H}^{-1} \textbf{b}. \end{aligned}$$

where:

- $\textbf{f}_c^\text {opt} = [f_{c,1}^\text {opt}, f_{c,2}^\text {opt}, \dots , f_{c,K}^\text {opt}]^T$ is the vector of control amplitudes.
- $\textbf{H}$ is a Hermitian matrix of size $K \times K$, with elements:
  14 $$\begin{aligned} H_{k,j} = q_{r,\text {unit},k}^H \mathbf {R(\omega )} q_{r,\text {unit},j} + \frac{1}{\rho _a c} A_s q_{p,\text {unit},k}^H q_{p,\text {unit},j}. \end{aligned}$$
- $\textbf{b}$ is a vector of size *K*, with elements:
  15 $$\begin{aligned} b_k = -\Big ( q_{r,\text {unit},k}^H \mathbf {R(\omega )} q_{r,\text {pri}} + \frac{1}{\rho _a c} A_s q_{p,\text {unit},k}^H q_{p,\text {pri}} \Big ). \end{aligned}$$

In this study, three actuators are considered ($K=3$). Accordingly, the system of equations can be expressed as

16 $$\begin{aligned} \begin{aligned}&H_{1,1} f_{c,1}^\text {opt} + H_{1,2} f_{c,2}^\text {opt} + H_{1,3} f_{c,3}^\text {opt} = -b_1, \\&H_{2,1} f_{c,1}^\text {opt} + H_{2,2} f_{c,2}^\text {opt} + H_{2,3} f_{c,3}^\text {opt} = -b_2, \\&H_{3,1} f_{c,1}^\text {opt} + H_{3,2} f_{c,2}^\text {opt} + H_{3,3} f_{c,3}^\text {opt} = -b_3. \end{aligned} \end{aligned}$$

Solving this system yields the optimal gains $f_{c,\text {opt},1}$, $f_{c,\text {opt},2}$, and $f_{c,\text {opt},3}$. A genetic algorithm^3^ is then used to search for the actuator locations that minimize the system’s acoustic response, with the search space defined by the possible positions of the three actuators. It should be highlighted that this is a theoretical calculation of the sound power, and it would be difficult to obtain one-to-one validation with real control setup. However, this method offers a valuable guidance for identifying optimal actuator locations to achieve effective noise reduction, as discussed in the Results section.

**Fig. 2:**
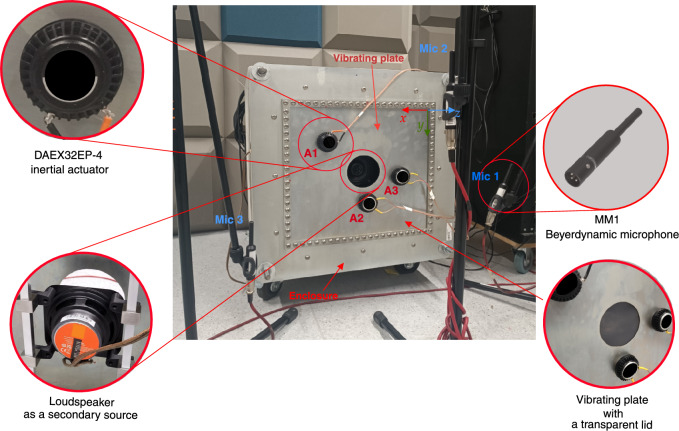
Overview of the experimental rig showing a concrete enclosure with a vibrating plate that contains a central circular opening of radius 0.05 m. The steel plate spans 0.42 m in *x*, 0.39 m in *y*, and has a thickness of 0.001 m in $-z$. Actuators (A1, A2, and A3) are mounted on the plate at (0.32 m, 0.1 m), (0.2 m, 0.29 m), and (0.1 m, 0.2 m), respectively. Microphones (Mic 1, Mic 2, and Mic 3) are positioned at the coordinates (–0.18 m, 0.12 m, 0.25 m), (0.21 m, –0.1 m, 0.4 m), and (0.53 m, 0.24 m, 0.25 m), respectively. The loudspeaker, used as a secondary source, is centered behind the opening.

The following model properties are chosen due to their widespread use in various engineering applications and to reflect the experimental setup illustrated in Fig. 2. The plate is composed of steel with a density $\rho _p = 7850 \, \mathrm {kg/m^3}$, Young’s modulus $E = 210 \times 10^9 \, \textrm{Pa}$. The dimensions of the plate are $a = 0.42 \, \textrm{m}$, $b = 0.39 \, \textrm{m}$, and $h = 0.001 \, \textrm{m}$, representing its length, width, and thickness, respectively. The density of the actuator can be estimated based on its mass ($150\, \textrm{g}$) and geometric dimensions, assuming a radius of $0.02\, \textrm{m}$ and a fixed height of $0.001\, \textrm{m}$, which corresponds to the uniform thickness of the mixed interface between the plate and the actuators. The enclosure domain has a depth of $d = 0.4 \, \text {m}$, air density $\rho _a = 1.2 \, \text {kg/m}^3$, and speed of sound $c = 343 \, \text {m/s}$ with the primary noise source at (0.3 m, 0.1 m, -0.2 m). Using this system parameters, the optimal positions of the three inertial actuators were determined to be $(x = 0.32\, \text {m}, y = 0.10\, \text {m})$, $(x = 0.20\, \text {m}, y = 0.29\, \text {m})$, and $(x = 0.10\, \text {m}, y = 0.20\, \text {m})$. The resulting actuator positions are evaluated and compared with alternative actuator arrangements in the Results section, where the effectiveness of the analytical model as a tool for guiding the optimal placement of inertial actuators is discussed.

### Experiment

**Fig. 3:**
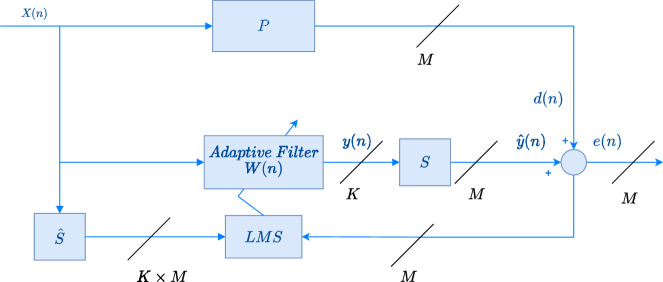
Block diagram of a single-reference multichannel feedforward FxLMS algorithm. $M$ and $K$ refer to the number of the output and secondary source, respectively.

The laboratory room measures 5.8 meters in length and 3.5 meters in width. While the walls are lined with sound-absorbing materials, the presence of equipment and other objects within the room gives it acoustic characteristics more representative

of a typical real-world environment rather than those of a dedicated acoustic chamber. The experimental setup consists of a rectangular steel plate mounted on a concrete enclosure, within which a loudspeaker is installed. The material and dimensions of the plate are as described in the Analytical model section. The concrete walls of the enclosure provide a high degree of sound attenuation; hence, the majority of the acoustic energy that exits the enclosure is transmitted through the plate and the circular opening. The loudspeaker, a Behringer EUROLIVE B208D with a rated maximum sound pressure level (SPL) of 113 dB, serves as the primary excitation source and is used to generate band-limited random noise. Three Beyerdynamic MM1 error microphones are used for the active control system with three DAEX32EP-4 inertial actuators. Although the actuator placement was optimized using theoretical sound power calculations, the experimental evaluation will be based on fixed microphone positions using adaptive active noise control. These positions are selected to capture the dominant acoustic field from the noise barrier, where the transmitted sound pressure is highest, and are spaced such that the distance between them, as well as their distance from the vibrating panel, remains small enough to allow for global noise reduction within the control region^26,42,43^. While variations in microphone placement may affect local details of the measured response, the overall global performance is not expected to be significantly influenced. A detailed investigation into the optimal placement of error microphones is considered beyond the scope of this study. For configurations incorporating a secondary loudspeaker source, a JBL Stage2 424 speaker is used and positioned centrally behind the opening, as in Fig. 2. To test the system with a reduced acoustic leakage effect, a plastic lid is adhesively bonded to the steel plate. The lid is made of semi-rigid polyvinyl chloride (PVC) with a density of approximately 1300 $\mathrm {kg/m^3}$. In this work, the Analog Device SC598 with SHARC+ core is used along with EVSOMCRR-EZKIT development board^44^. Thus, the system is equipped with the ADAU1979, a 4-channel, 24-bit Sigma-Delta ADC, and the ADAU1962, a 12-channel, 24-bit differential output DAC. For the selected ADC and DAC with a sampling frequency of 48 KHz, the group delay is 1 ms. Anti-aliasing IIR filters are designed for downsampling from 48 kHz to the downsampling frequency ($F_{s,D}$) of 3 kHz, followed by upsampling. Based on the analysis of the IIR filter characteristics, a 20-sample group delay is introduced into the system during both downsampling and upsampling, which is equivalent to 0.83 ms. Additionally, the system has a delay of 0.33 ms due to the output buffer handling. This makes the total latency of the system (from ADC to system to DAC) to be around 2.16 ms, which is borderline acceptable for active control implementation^6^. For active control, a feedforward multichannel FxLMS algorithm with a leakage factor is used due to its simplicity and robustness^6^, as illustrated in Fig. 3. Furthermore, the control system incorporates normalization based on the power of the single reference signal.

$X(n)$ represents the artificially generated reference signal. However, a reference microphone can be used if necessary. The primary path of the system is denoted by the matrix $P(n)$, which characterizes the acoustic propagation from the noise source to the error microphones. The active control filters are represented by the vector $W(n)$, consisting of $K$ adaptive FIR filters $W_k(z)$, each responsible for generating a component of the control signal vector $y(n)$. The vector $\hat{y}(n)$ represents the anti-noise signals applied through the actuators. The matrix $S(n)$, which models the secondary path dynamics between actuators and microphones, is generally not accessible in real-time operation and is therefore substituted with its estimate $\hat{S}(n)$. The $K\times M$ secondary paths of the plant is modeled as the impulse response of FIR filters using the normalized Least Mean Square (NLMS) algorithm^45^. This algorithm updates the coefficients of the K adaptive filters $W_{k}(n)$ in the controller as follows:

17 $$\begin{aligned} W_{k}(n+1) = (1 - \mu _{n}(n) \beta ) W_{k}(n) + \mu _{n}(n) \sum _{m=1}^{M} {\textbf {x}}'_{km}(n)e_m(n), \end{aligned}$$

where ${\textbf {x}}'_{km}(n)$ is the filtered reference signal, $e_m(n)$ is the error signal, and $\mu _{n}(n)$ is the normalized step size given by

18 $$\begin{aligned} \mu _{n}(n) = \frac{\mu }{\sum _{k=1}^{K}\sum _{m=1}^{M} \Vert x'_{km}(n)\Vert ^2}, \end{aligned}$$

where $\mu$ is the fixed step size, $\Vert \cdot \Vert$ represents the $L^2$-norm and $\beta$ is a regularization factor that applies a leakage effect.

## Results

**Fig. 4:**
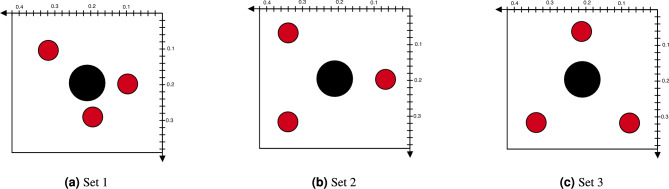
(**a**) Set 1: Actuators positioned based on the analytical model. (**b**) Set 2: Actuators localized toward the left half of the plate. (**c**) Set 3: Positioned with a similar concentration toward the bottom half of the plate.

**Fig.5:**
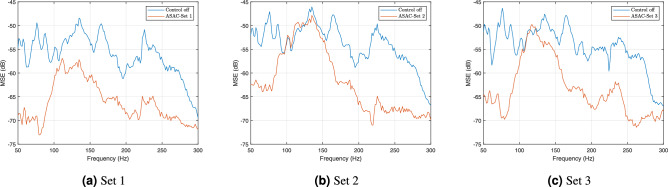
Experimental mean squared error (MSE) at the microphones for (**a**) Set 1, (**b**) Set 2, and (**c**) Set 3 actuator arrangements, with and without applying ASAC.

The optimal locations for the inertial actuators, as identified in the Methods section, are assessed using the ASAC approach and compared against alternative actuator arrangements as illustrated in Fig. 4. Experimental investigation of the three actuator sets demonstrates that Set 1 yields the best performance, as indicated by the mean squared error (MSE) measured at the three microphones and illustrated in Fig. 5. In passive configurations, placing actuator masses near the opening reduces MSE below 200 Hz but leads to increased peaks between 200 and 300 Hz (Fig. 5a ). This behavior is attributed to the opening’s location, which coincides with regions of high modal displacement at low frequencies^3^. Adding mass in this region increases the local inertia and alters the modal participation of these modes, making them less efficiently excited^46^. If a purely passive solution is required, the model can be adapted by eliminating the actuator forces and optimizing the locations of the added masses. However, this approach is primarily effective for shifting acoustic resonances from one frequency band to another. In such cases, the cost function may be modified to target narrowband noise reduction objectives^38^. This change leads to a reduction in vibration energy near the opening and thus a decrease in the MSE associated with sound radiation at lower frequencies. When ASAC is applied, Set 1 consistently yields the lowest MSE across nearly the entire frequency range. In the 100–200 Hz band, it achieves an average reduction approximately 5 dB greater than that obtained with Sets 2 and 3. This frequency range is particularly critical, as it corresponds to dominant the acoustic leakage through the opening, which is discussed further in this section. The placement of actuators in Set 1, near the edge of the opening, facilitates a greater displacement range and thus enhances control effectiveness. In contrast, Sets 2 and 3 with actuators positioned near the clamped edges of the plate, where higher structure stresses result in elevated resonance levels and limited displacement. These factors collectively reduce their capacity to control vibrations, suppress radiated sound, and generate effective anti-noise signals. These findings indicate the efficacy of the proposed analytical model in guiding optimal actuator placement for improved noise reduction.

**Fig. 6:**
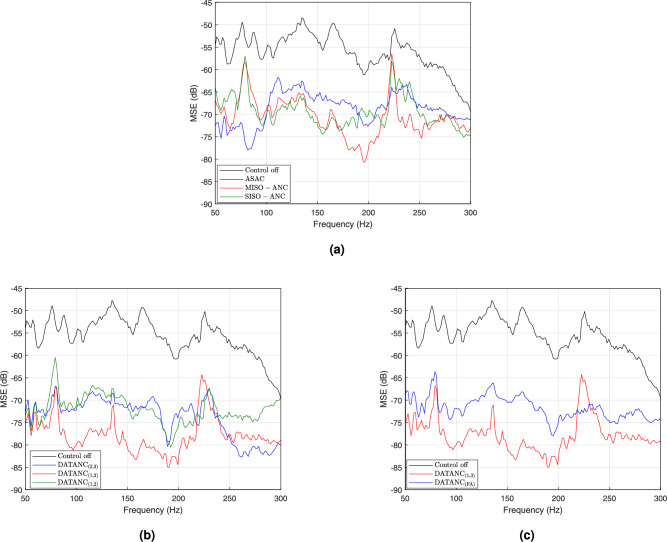
MSE performance comparison across different active noise control configurations. (**a**) Conventional ANC methods: ASAC (three inertial actuators), SISO-ANC (a single loudspeaker with one error microphone), and MISO-ANC (a loudspeaker with three microphones). (**b**) Proposed DATANC configurations in which individual inertial actuators are selectively deactivated while the secondary loudspeaker remains active. (**c**) Comparison between $\mathrm {DATANC_{(1,3)}}$, which uses the loudspeaker along with actuators A1 and A3, and $\mathrm {DATANC_{(FA)}}$, which includes all actuators.

Based on this optimization, the inertial actuators’ arrangement in Set 1, also shown in the experimental setup (Fig. 2), is hereafter referred to as the ASAC configuration throughout this work. To assess the performance of conventional active control strategies, comparative experiments are conducted under identical acoustic and structural conditions. The enclosure was excited using a loudspeaker generating band-limited white noise in the 50–300 Hz range. The adaptive control algorithm employs control filters of length 400 and secondary path models of length 300, with a step size of $\mu = 1 \times 10^{-6}$ and a regularization factor of $\beta = 1 \times 10^{-6}$. These parameters were experimentally tuned and remain fixed across all control configurations unless stated otherwise. The first configuration, ASAC, employs three error microphones and three inertial actuators, arranged according to the optimized layout. The second, Multiple-Input Single-Output Active Noise Control (MISO-ANC), follows a conventional overdetermined approach using a single loudspeaker as the actuator with three error microphones. The third, Single-Input Single-Output Active Noise Control (SISO-ANC), represents a single-channel scheme using Mic 2 as the error microphone, while Mic 1 and 3 are used only as observation microphones. In the frequency range below 100 Hz, the ASAC configuration exhibits lower MSE values compared to the loudspeaker-based systems, as in Fig 6a . However, this band is particularly challenging to control, as it contains the system’s fundamental natural frequencies^3^. Moreover, this frequency range is generally less critical in practice, as sounds below 100 Hz tend to have lower perceptual impact, particularly at moderate listening levels^47,48^. While the methodology proposed in this work can be extended to different frequency ranges, the selected range remains relevant for various engineering applications, such as noise control casings and household appliances^2,24,49^, where dominant noise energy is often concentrated above 100 Hz, making attenuation in this band more critical than at lower frequencies. The secondary loudspeaker in the MISO-ANC and SISO-ANC configurations is also less effective in this band, with a typical lower operating limit near 65 Hz. Thus, although ASAC shows better performance at this range, the perceptual and practical impact of this advantage remains limited. In the frequency range dominated by the acoustic leakage through the opening (100–200 Hz), the loudspeaker-based configurations (MISO-ANC and SISO-ANC) demonstrate advanced performance compared to the ASAC configuration.

This improvement is due to the reduced vibro-acoustic coupling efficiency of the system in this band. Nevertheless, ASAC achieves an average noise reduction of approximately 12 dB, which remains significant for applications where only inertial actuators can be used. Loudspeaker-based configurations, however, achieve greater reduction in this frequency range, with MISO-ANC outperforming SISO-ANC by more than 2 dB on average. This additional performance comes at the cost of higher computational complexity, as MISO-ANC processes multiple reference signals and adapts multiple control paths simultaneously. Notably, despite relying on a single error microphone, the SISO-ANC configuration demonstrates a considerable global noise reduction across all three error microphones (for microphones power spectrograms see Supplementary information). A distinct resonance near 225 Hz, corresponding to a natural frequency of the system dominated more by the vibro–acoustic coupling, is observed due to the excitation from the primary source. As shown in Fig. 6a , loudspeaker-based configurations exhibit limited control performance at this frequency. This discrepancy is due to the placement of the loudspeaker near the enclosure opening, which limits its ability to effectively excite the structural modes. Consequently, the secondary source shows weak coupling with the vibrating structure, reducing its effectiveness in canceling the noise associated with this dominant resonance. The analysis of the system response using conventional control methods reveals that different actuator types exhibit superior performance in distinct frequency bands; hence, a hybrid approach that combines inertial and loudspeaker actuators may offer enhanced broadband control. This approach, referred to as Dual-Actuator-Type Active Noise Control (DATANC), is proposed based on insights from the performance of conventional control methods. The initial DATANC implementation employs a fully determined configuration with three error microphones and a combination of two inertial actuators and one loudspeaker, where actuator A1, A2, or A3 is deactivated in separate setups. These configurations are denoted as $\mathrm {DATANC_{(2,3)}}$, $\mathrm {DATANC_{(1,3)}}$, and $\mathrm {DATANC_{(1,2)}}$, respectively, with the deactivated actuator remaining physically present. Their system responses are presented in Fig.6b . The underdetermined configuration ($\mathrm {DATANC_{(FA)}}$) uses all four actuators simultaneously to evaluate performance under increased actuation power, as shown in Fig.6c .

### Dual-actuator-type active noise control

**Fig. 7:**
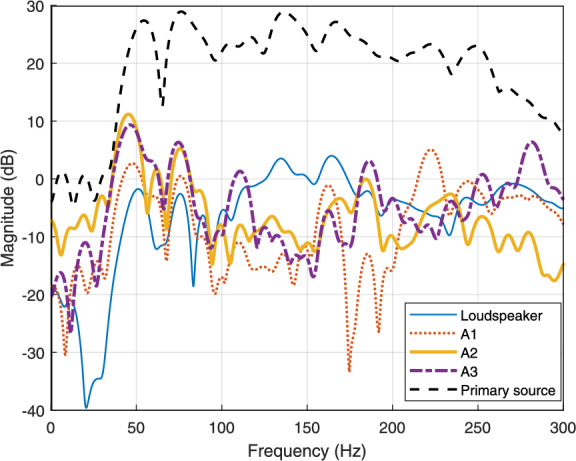
Average magnitude responses of the acoustic transfer functions from the primary source and each secondary actuator (Loudspeaker, A1, A2, A3) to the microphones. The responses are averaged across the three microphones.

Among the fully determined DATANC configurations, $\mathrm {DATANC_{(2,3)}}$ and $\mathrm {DATANC_{(1,2)}}$ exhibit acceptable performance in the 100–200 Hz frequency range, achieving an average MSE reduction of approximately 18 dB across the error microphones. This level of attenuation is comparable to that obtained with the MISO-ANC configuration, as shown in Fig. 6b . However, $\mathrm {DATANC_{(1,3)}}$ outperforms both configurations, providing over 8 dB of additional MSE reduction relative to $\mathrm {DATANC_{(2,3)}}$ and $\mathrm {DATANC_{(1,2)}}$ within the same frequency band. The spatial proximity between the loudspeaker and actuator A2 near the enclosure opening, negatively affects their mutual coherence. Thus, when both sources are active simultaneously, their acoustic fields interact destructively in this frequency band. This phenomenon is also observed in the $\mathrm {DATANC_{(FA)}}$ configuration, as in Fig. 6c . The resonance observed around 225 Hz lies outside this band. As a result, it leads to a reduction of more than 20 dB compared to the scenario without active control. This spatial analysis of the relative positions of the loudspeaker, inertial actuators, and vibrating plate is reflected in the transfer functions of the primary and secondary paths associated with the four actuators. These paths are identified using the normalized least mean square (NLMS) algorithm. Fig. 7 shows the average magnitude response of the primary and secondary paths across the three microphones.

**Fig. 8:**
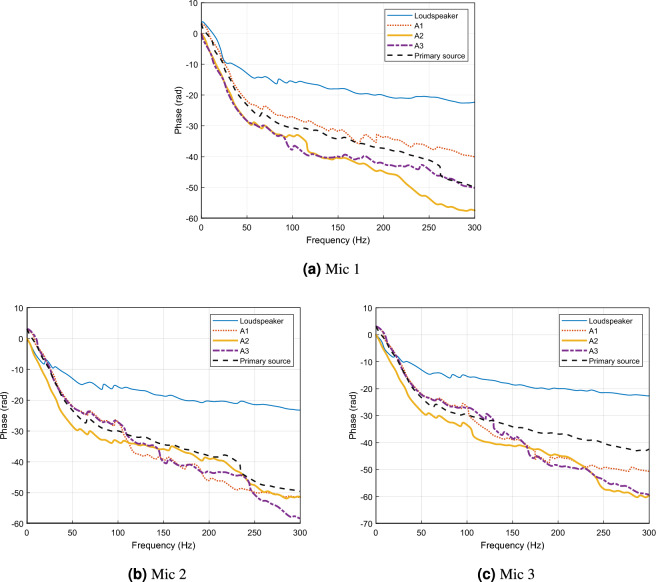
Phase responses of the acoustic transfer functions from each secondary source (Loudspeaker, A1, A2, A3) and the primary noise source to the three microphones: (**a**) Mic 1, (**b**) Mic 2, and (**c**) Mic 3.

The secondary loudspeaker exhibits the highest transfer function gain in the 100–200 Hz range, outperforming the inertial actuators. This higher response highlights the loudspeaker’s dominant role in compensating for acoustic leakage through the enclosure opening. A3 demonstrates superior magnitude performance over the other inertial actuators, particularly in the 100–120 Hz and 180–200 Hz bands, making it a strong candidate for effective noise reduction within this frequency range (see Supplementary information for individual path responses).

**Fig. 9:**
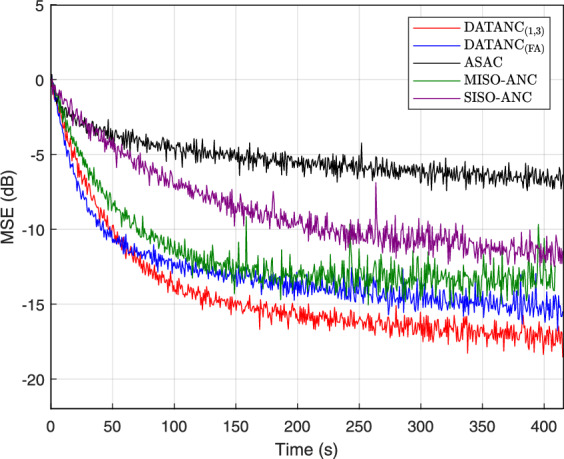
MSE comparison over time for the conventional and the proposed active control configurations.

In the phase responses shown in Fig.8a and Fig.8c , the paths associated with actuator A2 at Mic 1 and Mic 3 exhibit the greatest mismatch, not only with the primary path but also relative to the other secondary paths. A2 also shows an abrupt deviation from its prior phase trend beyond 100 Hz. This irregular and weakly correlated phase behavior reduces overall phase consistency across the control channels, thereby limiting the coherence required for effective noise cancellation in the $\mathrm {DATANC_{(FA)}}$ configuration. To summarize, the superior noise reduction performance of $\mathrm {DATANC_{(1,3)}}$ can be explained by the spatial arrangement of the actuators and the characteristics of their transfer paths: $\mathrm {DATANC_{(1,3)}}$ avoids destructive spatial coupling and benefits from more consistent phase relationships across the control channels, which together account for its superior attenuation performance. As shown in Fig. 9, the $\mathrm {DATANC_{(FA)}}$ configuration demonstrates the fastest convergence rate among all tested configurations. This rapid adaptation is due to the additional degrees of freedom provided by the underdetermined structure. However, this improvement comes at the cost of increased computational complexity, as more channels are used and solve an underdetermined system during adaptation. $\mathrm {DATANC_{(1,3)}}$ configuration also performs better than the conventional control methods, achieving both faster convergence and a lower steady-state error.

### Acoustic leakage

**Fig. 10:**
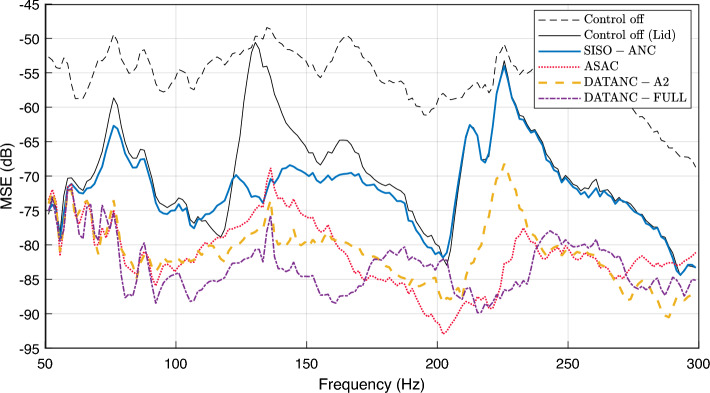
MSE performance for different active noise control configurations in the presence of a structural opening sealed with a transparent lid. “Control Off” denotes the baseline configuration with the opening, while “Control Off (Lid)” indicates the same system with the lid applied. For ASAC, parameters were set to $\mu = 1 \times 10^{-6}$ and $\beta = 1 \times 10^{-6}$; for all other configurations, $\mu = 1 \times 10^{-7}$ and $\beta = 1 \times 10^{-5}$.

To assess the contribution of the sound transmission through the opening to the overall system response, the system is tested using a plate structure with a transparent plastic lid in the place of the former opening. In practice, this also beneficial for enclosures where a view of the interior is required, or where a different material is needed for part of the wall due to various reasons (e.g., casings of household appliances often use different materials). As shown in Fig. 10, the passive system response highlights the impact of the structural opening: without the lid (Control off), the average sound pressure level is approximately -56 dB, whereas with the lid in place (Control off (Lid)), it is reduced to around -70 dB, clearly demonstrating the effect of sound transmission through the opening. In the 100–200 Hz band, the loudspeaker-based configuration (SISO-ANC) no longer outperforms the ASAC approach in the presence of the transparent lid. For reference, see Fig. 6a for the corresponding comparison in the case of the system with acoustic leakage.

**Fig. 11:**
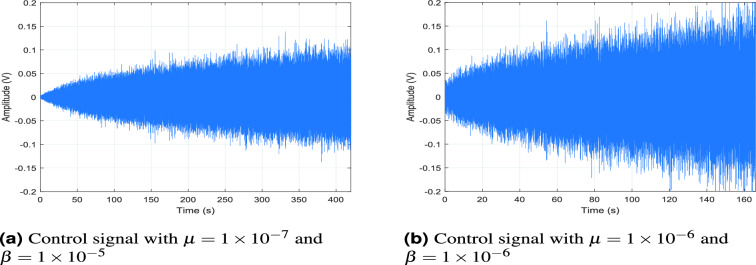
Control signals for the actuator in a single-channel ANC system with a transparent lid. Panels (**a**) and (**b**) show signals generated using $\mu = 1 \times 10^{-7}$, $\beta = 1 \times 10^{-5}$, and $\mu = 1 \times 10^{-6}$, $\beta = 1 \times 10^{-6}$, respectively.

The ASAC system achieves over 30 dB attenuation at certain frequencies and an average reduction of approximately 12 dB across this frequency range, compared to only 4 dB for the SISO-ANC configuration. Additionally, the previously observed destructive acoustic coupling between inertial actuator A2 and the loudspeaker is no longer present, which enhances the performance of the full actuation configuration ($\mathrm {DATANC_{(FA)}}$), yielding an average reduction of 15 dB compared to 12 dB for $\mathrm {DATANC_{(1,3)}}$. At higher frequencies, particularly around 225 Hz where the dominant vibro-acoustic resonance of the system occurs, the SISO-ANC configuration achieves no or minimal noise reduction. This underscores its limited effectiveness in addressing strongly coupled vibro-acoustic behavior at those frequencies. This limitation in performance also points to the need for further parameter tuning in the SISO-ANC configuration. In the absence of acoustic leakage, the actuator’s ability to influence the acoustic field is reduced, making the system more sensitive to the adaptation parameters. For example, the SISO-ANC setup becomes unstable after 166 s when using $\mu = 1 \times 10^{-6}$ and $\beta = 1 \times 10^{-6}$, whereas improved stability is observed with $\mu = 1 \times 10^{-7}$ and $\beta = 1 \times 10^{-5}$, as shown in Fig. 11.

## Discussion

This study investigates the performance of various active noise control strategies in vibro-acoustic systems that include openings in their noise barriers. Such systems are common in engineering applications where additional functions, such as airflow, are required. The analysis focuses on both conventional Active Noise Control (ANC) and Active Structural Acoustic Control (ASAC) approaches.A modification of a vibro-acoustic analytical model is proposed to account for sound transmission through the opening. Consequently, the optimization of the inertial actuator placements is guided by both sound radiation and acoustic leakage, demonstrating reliable guidance for actuator arrangement that captures the key physical characteristics of the system and results in more effective ASAC performance in experimental testing compared to alternative configurations. It also analyzes the proposed approach, Dual-Actuator-Type Active Noise Control (DATANC), which integrates the advantages of loudspeakers and inertial actuators. Among the tested ASAC configurations, the setup with actuators positioned near the edge of the structural opening (Set 1) achieves the lowest MSE, with an average reduction of 12 dB in the 100–200 Hz band. In comparison, Sets 2 and 3 achieve average reductions of 5 dB and 8 dB, respectively. This enhanced performance is attributed to the increased local inertia from the added mass and the actuators’ ability to induce greater structural displacement in the vibrating plate. Sound transmission through the system’s structural opening predominantly affects the 100–200 Hz frequency range. This behavior is confirmed experimentally by evaluating the system’s performance with and without a transparent PVC lid used to minimize acoustic leakage. As shown in Table 1, the passive configuration with the lid (Control off (Lid)) achieves a substantially lower average MSE of –68.61 dB, compared to –53.90 dB for the open configuration (Control off).

**Table 1:** Summary of performance metrics across passive and active control configurations for the system with an opening.

Configuration	Average MSE [100–200 Hz] (dB)	MSE near 225 Hz (dB)	MPI
Control off	−53.90	−50.84	–
$\mathrm {DATANC_{(1,3)}}$	**−79.73**	−64.28	9900
$\mathrm {DATANC_{(1,2)}}$	−71.94	−67.30	9900
$\mathrm {DATANC_{(2,3)}}$	−71.48	−67.45	9900
MISO−ANC	−71.32	−56.47	3300
$\mathrm {DATANC_{(FA)}}$	−70.84	**−70.83**	13200
ASAC	−66.37	−63.31	9900
SISO−ANC	−65.08	−58.20	1100
Control off (Lid)	−68.61	−50.58	–

The reported average MSE values are first computed as the mean across the three microphones, and then averaged over the 100–200 Hz frequency band. The MSE near 225 Hz corresponds to the dominant vibro-acoustic resonance of the system. MPI (Multiplications Per Iteration) indicates the computational complexity required for each control update.

Thus, in the 100–200 Hz frequency range where sound transmission through the opening dominates, loudspeaker-based control methods (MISO-ANC and SISO-ANC) outperform ASAC, which relies only on inertial actuators. However, inertial actuators are particularly effective at attenuating the system’s strong vibro-acoustic resonances, where this coupling coupling is high (e.g., around 225 Hz in the system under consideration). These observations highlight the need for a hybrid control strategy that integrates structural and acoustic actuation to ensure effective broadband attenuation. Nonetheless, such configurations must be carefully designed, as interaction between closely positioned actuators near the opening can degrade control performance. This is evidenced by the performance of $\mathrm {DATANC_{(1,3)}}$, which achieves better overall noise reduction than the full actuation configuration $\mathrm {DATANC_{(FA)}}$ while incurring lower computational cost. In contrast, $\mathrm {DATANC_{(FA)}}$ exhibits superior performance in reducing the resonance around 225 Hz, along with an enhanced convergence rate, due to the increased actuation availability. The computational complexity associated with each configuration by calculating the multiplications per iteration (MPI) using the formula $\text {MPI} = M_r \cdot P_a \cdot (2N_c + L_s)$, where $M_r$ denotes the number of input reference signals, $P_a$ represents the number of actuators or control signals, $N_c$ is the length of each control filter, and $L_s$ is the length of the secondary path model. The table also evaluates the computational complexity associated with each configuration by calculating the multiplications per iteration (MPI) using the formula $\text {MPI} = M_r \cdot P_a \cdot (2N_c + L_s)$, where $M_r$ denotes the number of input reference signals, $P_a$ represents the number of actuators or control signals, $N_c$ is the length of each control filter, and $L_s$ is the length of the secondary path model. Overall, this study underscores the importance of optimally placing the system actuators to enhance functionality, as demonstrated through analytical and experimental investigations. It also highlights the effectiveness of combining loudspeakers and inertial actuators for active noise control in systems with structural openings, offering a practical compromise between performance and computational cost. Future work will explore the application of DATANC in commercial appliances where lightweight panels and structural openings are essential for airflow, monitoring, and maintenance. These systems often suffer from noise leakage through necessary opening, making them ideal candidates for hybrid active control strategies that balance noise reduction with functional design constraints. Additionally, in more sophisticated systems, such as those requiring multiple openings and additional secondary loudspeakers, the flexibility of the analytical model and its proposed modifications will allow these configurations to be examined. Building on the current optimization framework, future investigations could establish relationships between plate size, the number of openings, and the required actuators to further inform practical design decisions.

## Supplementary Information

Supplementary Information.

## Data Availability

The datasets used and/or analysed during the current study available from the corresponding author on reasonable request.
