# Supplementary material for: Dual-actuator-type active noise control in vibro-acoustic systems with openings
Source: Sci Rep. 2025 Sep 2;15:32278. doi: 10.1038/s41598-025-17810-8 (PMC12402472; doi:10.1038/s41598-025-17810-8)
Supplement: Supplementary file 1 — Supplementary Information. [file 41598_2025_17810_MOESM1_ESM.pdf]

Dual-Actuator-Type Active Noise Control for Vibro-Acoustic Systems with Openings

Khaled Said Ahmed Maamoun[1,3,*], **Chung Kwan Lai**[2], **Stanislaw Wrona**[1], **Marek Pawelczyk**[1], **and Hamid Reza Karimi**[3]

[1]Silesian University of Technology, Department of Measurements and Control Systems, Gliwice, 44-100, Poland.
[2]University of Southampton, Institute of Sound and Vibration Research, Southampton, SO17 1BJ, United Kingdom.
[3]Politecnico di Milano, Department of Mechanical Engineering, Milan, 20156, Italy.
*Khaled.Maamoun@polsl.pl

Error signal performance at individual microphones

The schematic representation of the experimental setup is presented in **Supplementary Figure.** S1. It provides a three-dimensional overview of the enclosure, vibrating plate, and the positioning of the actuators as well as the microphones.

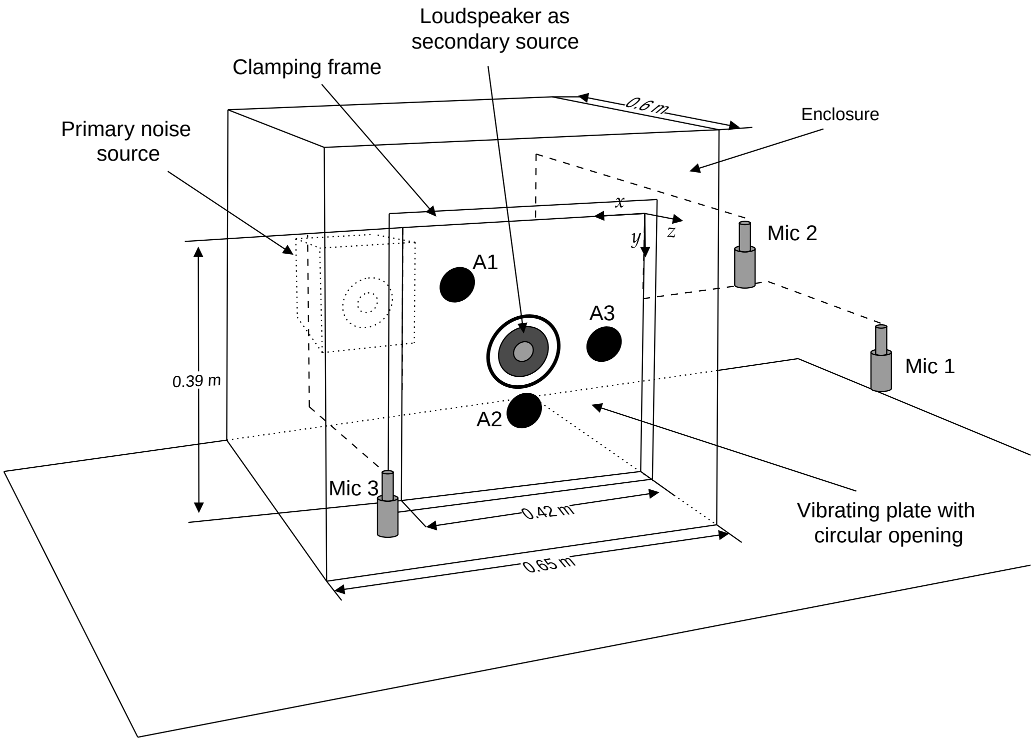

Supplementary Figure S1. Schematic of the experimental setup showing the enclosure with a vibrating plate that contains a central circular opening of radius 0.05 m. Inertial actuators (A1, A2, and A3) are mounted on the plate at (0.32 m, 0.1 m), (0.2 m, 0.29 m), and (0.1 m, 0.2 m), respectively. Microphones (Mic 1, Mic 2, and Mic 3) are positioned at the coordinates (-0.18 m, 0.12 m, 0.25 m), (0.21 m, -0.1 m, 0.4 m), and (0.53 m, 0.24 m, 0.25 m), respectively. The loudspeaker, used as a secondary source, is centered behind the opening, while the primary noise source is located inside the enclosure.

The modeling of the acoustic paths is performed using the Normalized Least Mean Square (NLMS) algorithm. The original signals are recorded at a sampling rate of 48 kHz and subsequently downsampled to 3 kHz to reduce computational complexity, given that the frequency content of interest lies below the Nyquist limit. A filter length of 300 coefficients is used for both the primary and secondary paths. The corresponding transfer functions, obtained from the measured impulse responses, are presented in **Supplementary Figure.** S2.

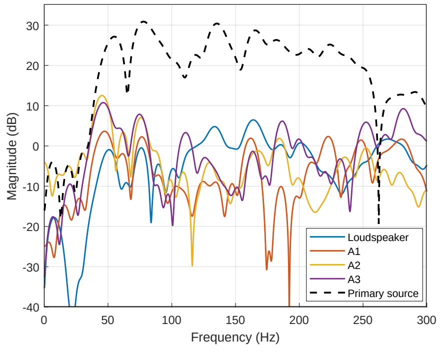

(a) Magnitude response of Mic 1.

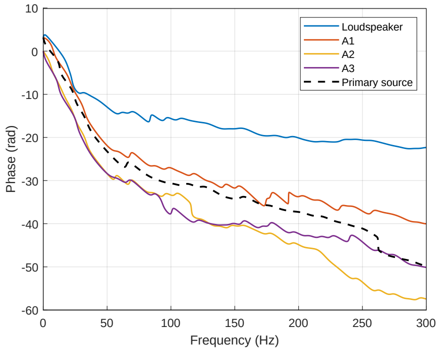

(b) Phase response of Mic 1.

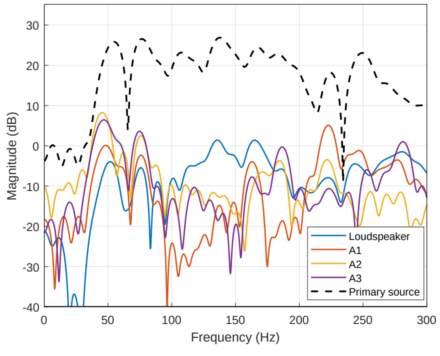

(c) Magnitude response of Mic 2.

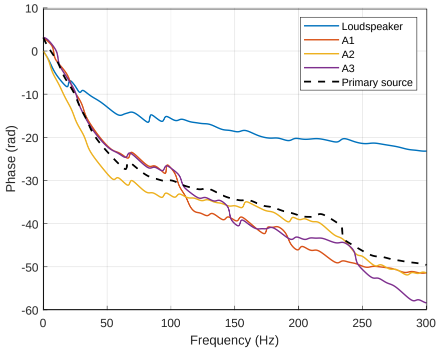

(d) Phase response of Mic 2.

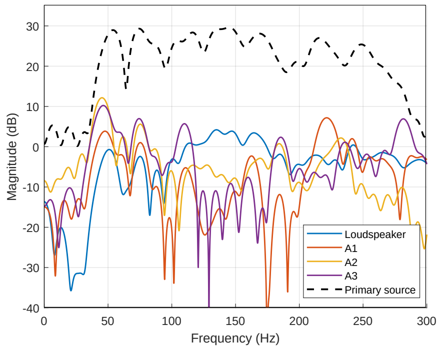

(e) Magnitude response of Mic 3.

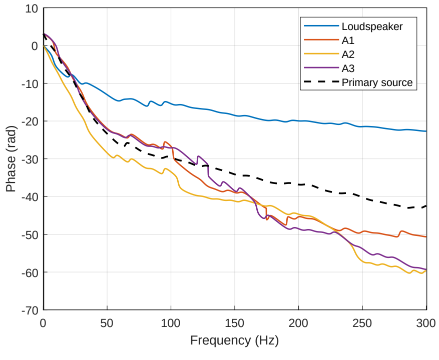

(f) Phase response of Mic 3.

Supplementary Figure S2. Frequency-domain analysis of the identified transfer functions for the three error microphones. Panels (a, b) show the magnitude and phase responses for Mic 1, respectively; panels (c, d) correspond to Mic 2; and panels (e, f) to Mic 3.

The concrete enclosure has five acoustically rigid boundaries, which reflect incident sound energy and minimize transmission through the walls. Consequently, the acoustic wave is predominantly transmitted through the vibrating plate and its central opening. To effectively capture the primary acoustic paths, the error microphones are deployed in a hemispherical configuration in the region in front of the vibrating plate. This spatial arrangement enhances observability of the pressure field and supports global noise reduction. This effectiveness is demonstrated by the performance of the Single-Input Single-Output Active Noise Control (SISO-ANC) scheme, which utilizes Mic 2 as the error sensor, while Mic 1 and 3 serve solely as observation sensors. Despite not being part of the control filter update, Mic 1 and Mic 3 exhibit significant reductions in acoustic power—comparable to their respective performances in the Multiple-Input Single-Output (MISO-ANC) configuration, as illustrated in **Supplementary Figure.** S3.

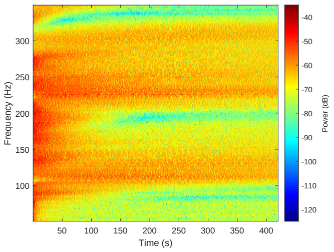

(a) Mic 1 with ASAC

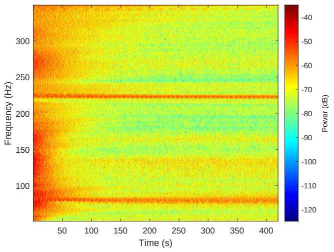

(b) Mic 1 with MISO-ANC

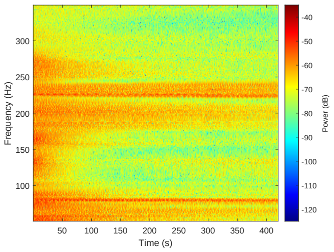

(c) Mic 1 with SISO-ANC

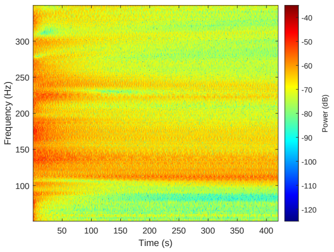

(d) Mic 2 with ASAC

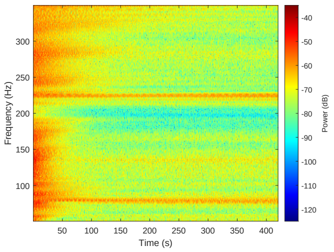

(e) Mic 2 with MISO-ANC

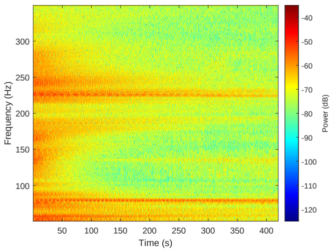

(f) Mic 2 with SISO-ANC

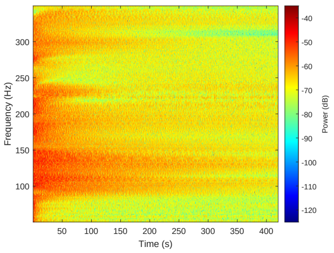

(g) Mic 3 with ASAC

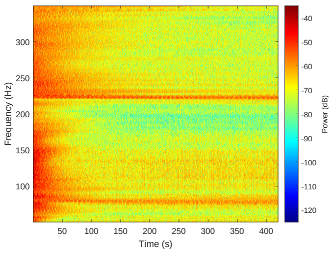

(h) Mic 3 with MISO-ANC

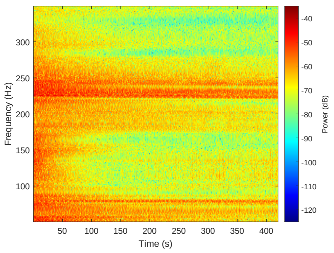

(i) Mic 3 with SISO-ANC

Supplementary Figure S3. Spectrograms showing microphone signal power over time. Panels **(a–c)** correspond to Mic 1 under Active Structural-Acoustic Control (ASAC), Multiple-Input Single-Output Active Noise Control (MISO-ANC), and Single-Input Single-Output (SISO-ANC), respectively; **(d–f)** correspond to Mic 2; and **(g–i)** to Mic 3 under the same control configurations. ASAC employs inertial actuators only, while MISO-ANC and SISO-ANC utilize the secondary loudspeaker as the only actuator.
